# Supplementary material for: An enzymatic cascade enables sensitive and specific proximity labeling proteomics in challenging biological systems
Source: Nat Commun. 2025 Nov 3;16:9691. doi: 10.1038/s41467-025-65405-8 (PMC12583597; doi:10.1038/s41467-025-65405-8)
Supplement: Supplementary file 1 — Supplementary Information [file 41467_2025_65405_MOESM1_ESM.pdf]

An enzymatic cascade enables sensitive and specific proximity labeling proteomics in challenging biological systems

Tommy J. Sroka[1,2], Lea K. Sanwald[1,#], Avishek Prasai[1,#], Josefine Hoeren[1,3], Valentina Trivigno[3], Valerie Chaumet[1,2], Louisa M. Krauß[1], Damian Weber[4], Daniela Yildiz[2,5,6,7,8], Karina von der Malsburg[1], Peter Walentek[4], Per Haberkant[9], Bianca Schrul[1,6,8], Kerstin Feistel[3] and David U. Mick[1,2,6,8,*]

1- Center for Molecular Signaling (PZMS), Department of Medical Biochemistry and Molecular Biology, Saarland University School of Medicine, Homburg, Germany
2- Center of Human and Molecular Biology (ZHMB), Saarland University School of Medicine, Homburg, Germany
3- Department of Zoology, Institute of Biology, University of Hohenheim, Stuttgart, Germany
4- Internal Medicine IV, Medical Center, CIBSS Centre for Integrative Biological Signalling Studies, SGBM Spemann Graduate School for Biology and Medicine, University of Freiburg, Germany
5- Experimental and Clinical Pharmacology and Toxicology, Molecular Pharmacology, Center for Molecular Signaling (PZMS), Saarland University, Homburg, Germany
6- PharmaScienceHub (PSH), Saarbrücken, Germany
7- Center for Gender-specific Biology and Medicine (CGBM), Saarland University, Germany
8- Center for Biophysics (ZBP), Saarland University, Germany
9- EMBL Heidelberg, Proteomics Core Facility, Heidelberg, Germany

[#], authors contributed equally

*, Correspondence: david.mick@uks.eu

SUPPLEMENTARY INFORMATION

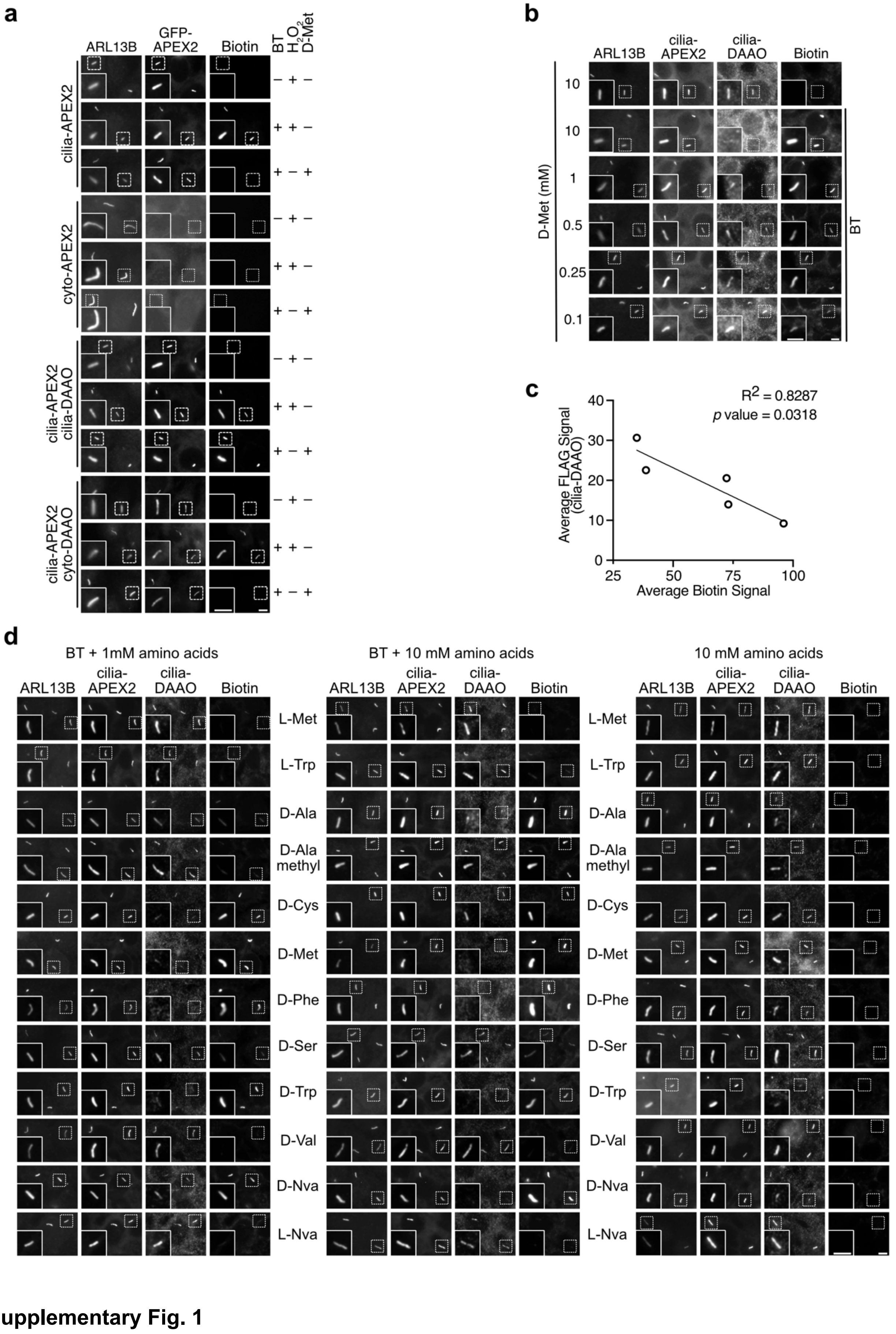

Supplementary Fig. 1

Supplementary Fig. 1: The DAAO-APEX2 enzymatic cascade requires co-localization and can use various D-amino acids as substrates.

(a) Representative immunofluorescence images of IMCD3 cells expressing cilia-APEX2, cilia-iAPEX and the cytosolic controls, cyto-APEX and cyto-DAAO. Cells were incubated with biotin tyramide (BT) for 30 min and either with H_2O_2 for 3 min for direct activation of APEX2, or with D-Met for 30 min for local production of H_2O_2 by DAAO as indicated. ARL13B antibody was used to stain for primary cilia. GFP fluorescence marked APEX2. Biotin was detected by fluorescent streptavidin.

(b) Proximity biotinylation of FLAG epitope precludes antibody binding. Proximity labeling in cilia-iAPEX IMCD3 cells was performed with indicated D-Met concentrations and cells analyzed by immunofluorescence microscopy, using fluorescently labeled streptavidin to detect biotin, anti-FLAG antibodies to detect cilia-DAAO, and anti-ARL13B antibodies to detect cilia. GFP fluorescence visualizes cilia-APEX2.

(c) FLAG and biotin signals from micrographs as shown in (b) have been quantified and average signals from random cilia (n = 99) plotted, followed by regression analysis. Source data are provided as a Source Data file.

(d) Representative immunofluorescence images of D-amino acid-dependent proximity labeling in IMCD3 cells expressing cilia-iAPEX. Various D- and L-amino acids were incubated at 1 or 10 mM concentration together with or without 500 μM BT for 30 min. Nva, norvaline. ARL13B antibody was used to stain for primary cilia. GFP fluorescence marked APEX2. FLAG antibody marked cilia-DAAO. Biotin was detected by fluorescent streptavidin.

All scale bars = 5 μm.

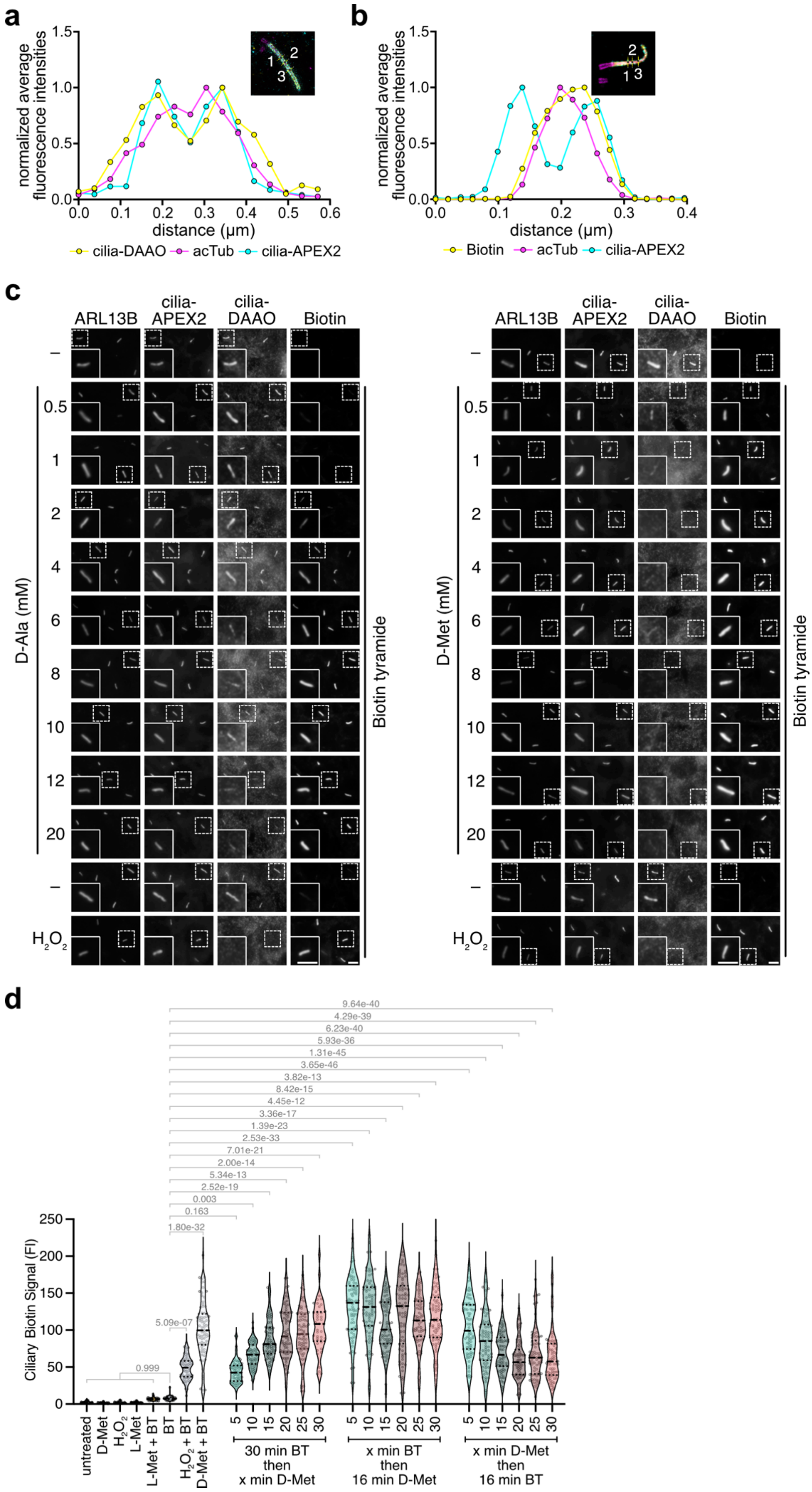

Supplementary Fig. 2

Supplementary Fig. 2: DAAO in vicinity of APEX2 allows spatiotemporal proximity labeling with amino acid substrate and concentration dependence.

(a) and (b) Line plots show the fluorescence signal intensities of individual channels of the U-ExM confocal images presented in **Fig. 2a** and **2b**, respectively. Shown are average signal intensities of three lines normalized to the maximum signal intensity in the respective channels, as indicated in the insets. **(a)** Line plots indicate localization of cilia-DAAO (yellow) and cilia-APEX2 (cyan) to the ciliary membrane surrounding the axoneme (marked by acTub in magenta). **(b)** Line plots show biotin signals (yellow) after iAPEX labeling in relation to the cilia membrane (marked by cilia-APEX2 in cyan) and the axoneme (acTub in magenta).

(c) The iAPEX-based biotinylation relies on DAAO-mediated oxidative deamination and is substrate and concentration dependent. Representative immunofluorescence micrographs of DAAO substrate titration in IMCD3 cells stably expressing cilia-iAPEX (quantification shown in **Fig. 2c**). APEX labeling was performed by incubating cells for 30 min with 500 µM biotin tyramide (BT) together with varying concentrations (range from 0.5-20 mM) of D-alanine or D-methionine or 1 mM H_2O_2 (for 2 min). Antibody staining against ARL13B labeled primary cilia, while staining against FLAG marked cilia-DAAO. GFP fluorescence visualized cilia-APEX2, and biotin was detected by fluorescent streptavidin.

(d) Pre-incubation with BT increases (left) while pre-incubation with D-Met lowers APEX2 activity (right). Violin plots show quantified ciliary biotin signals. APEX labelings were performed by incubating cells for different times and in different orders with 500 µM BT and 10 mM D- or L-Met. Quartiles and median are indicated by dotted and dashed lines, respectively. n = 77 cilia per condition. Data were analyzed using one-way ANOVA with Dunn's multiple comparisons.

All scale bars = 5 µm.

Source data are provided as a Source Data file.

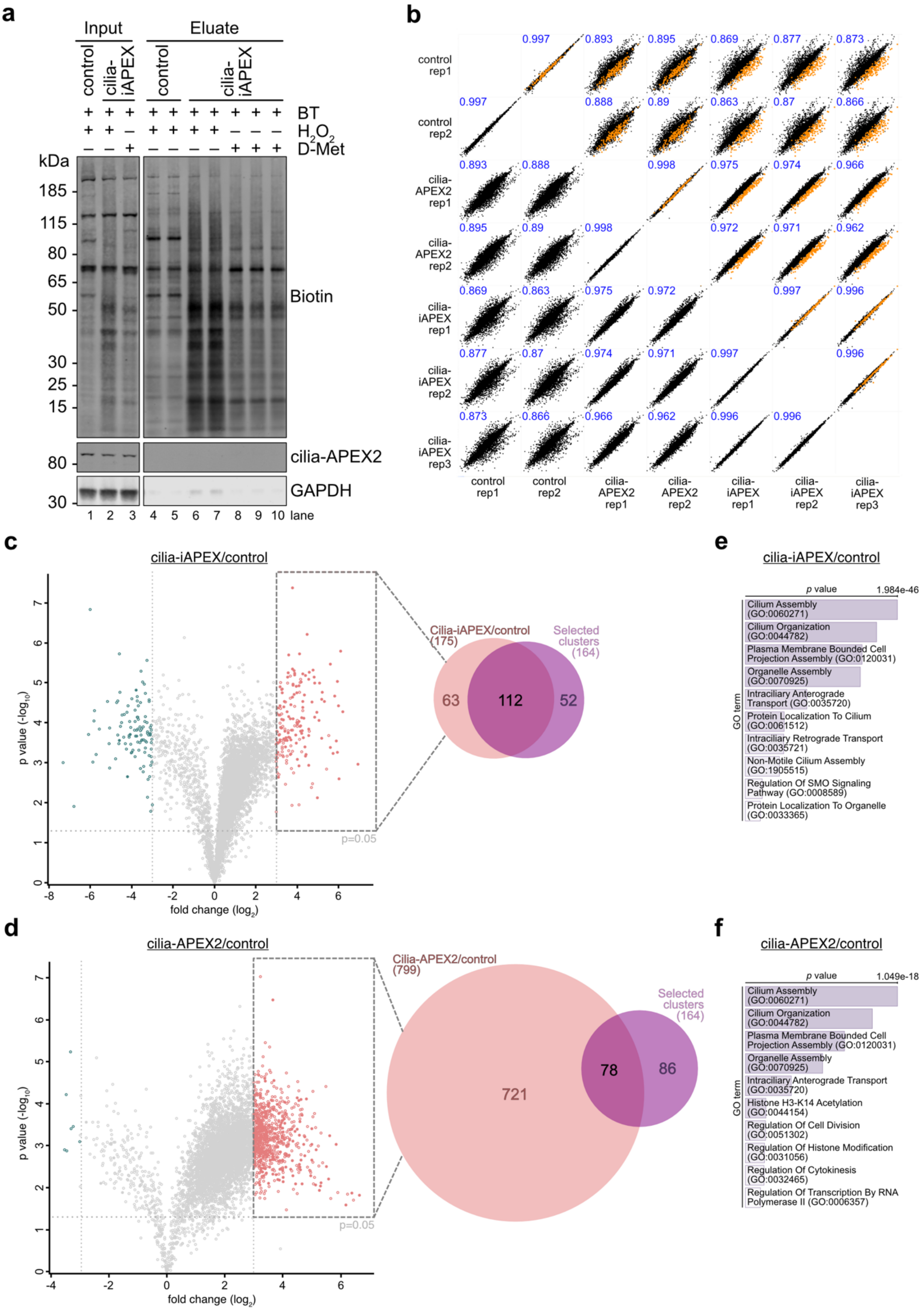

Supplementary Fig. 3

Supplementary Fig. 3: Hierarchical clustering of cilia-iAPEX proteomics data shows higher sensitivity and specificity than enrichment analysis.

(a) Western blot analysis of samples after proximity labeling from IMCD3 cilia-iAPEX or cilia-ablated cilia-APEX2 *Cep164*$^{-/-}$ cells (control), as outlined in (**Fig. 4a**) (n = 2 independent experiments; see **Fig. 4b**). Input and Eluate samples were separated by SDS-PAGE and analyzed by western blotting. Biotin was detected by fluorescently labeled streptavidin, cilia-APEX2 by antibodies against GFP. Input 0.063 %, Eluate 1.5 %. Stronger biotinylation was observed after H_2O_2-induced labeling.

(b) All identified proteins in the proteomics dataset from IMCD3 cells were analyzed by pairwise multiscatter plots, generated in Perseus using $\log_2$-transformed, imputed and Z-Score normalized protein abundance values (see **Supplementary Data 1**). Pearson correlation coefficients shown in blue. Top right, ciliary cluster hits (see **Fig. 4d**) are highlighted in orange.

(c and d) Volcano plots display statistical significance *versus* protein enrichment of cilia-iAPEX2 **(c)** and cilia-APEX2 **(d)** proteomics compared with control samples. *p* values (unpaired Student's *t* test) and TMT ratios were calculated from duplicate samples and plotted for 5982 proteins. Proteins are indicated by grey circles. Proteins with TMT ratios $>2^3$ and $<2^3$ are indicated by red and blue circles, respectively. Filled circles mark cilia proteins identified in May *et al.*, 2021. Venn diagrams show numbers of enriched proteins (TMT ratios $>2^3$) and overlap with selected cilia protein clusters from IMCD3 cells (see **Fig. 4d**)

(e) GO term enrichment analysis of cilia-iAPEX candidate proteins from (c) shows highly significant enrichment of proteins associated with cilia processes, including SHH signaling and protein trafficking.

(f) GO term enrichment analysis of proteins enriched in cilia-APEX2 samples (d) shows lower *p* values and enrichment of non-ciliary categories. *p* values were calculated by Fisher's exact test.

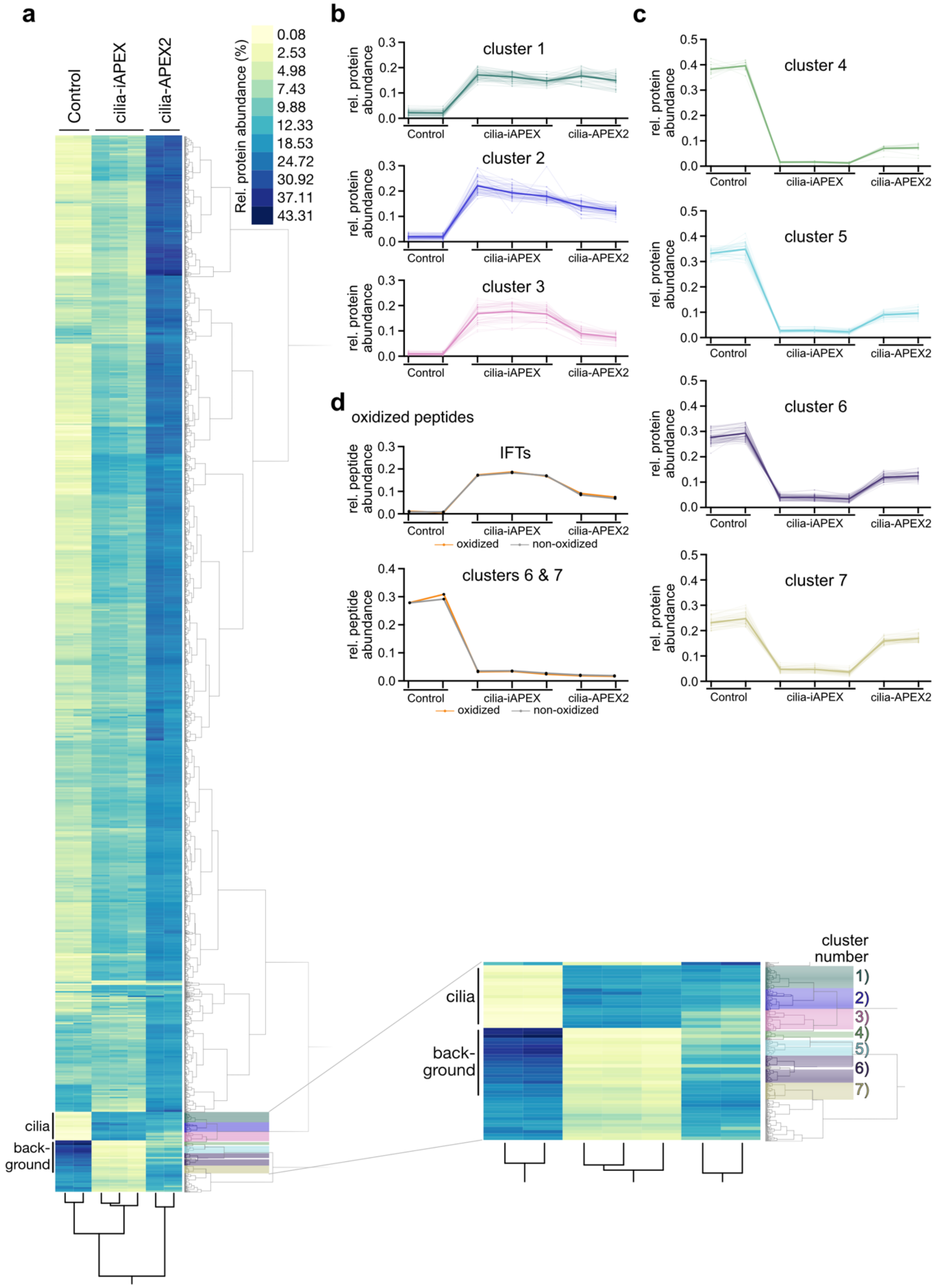

Supplementary Fig. 4

Supplementary Fig. 4: Hierarchical clustering of cilia-iAPEX proteomics in IMCD3 cells identifies cilia proteins and false-positive hits of previous studies.

(a) Relative protein abundances (rows) of the individual samples (columns) from IMCD3 cilia-iAPEX proteomics experiment (see **Fig. 4a**) were analyzed by two-way hierarchical clustering (Ward's method). Relative abundance of each protein was determined by dividing its individual TMT signal by the sum of TMT signals across all samples. The color legend for relative abundances (in %) is displayed. All quantified proteins are shown. Clusters containing cilia proteins, as well as example background clusters were highlighted. Cilia clusters (b) and background clusters (c) are shown in magnified views.

(b and **c)** Line plots show the relative abundances of each protein across samples in the cilia clusters **(b)** and selected background clusters **(c)**. The average abundances of all proteins within the clusters are represented by thick lines, individual proteins by thin lines.

(d) Relative abundances of oxidized (orange) and non-oxidized (black) peptides across samples among IFT proteins, exemplifying known cilia proteins (top), and proteins in clusters 6 and 7 (bottom). Only peptides of proteins, for which oxidized and non-oxidized peptides were identified, were analyzed. Averages of 55 oxidized and 596 non-oxidized peptides for IFT proteins, and 48 oxidized and 649 non-oxidized peptides for proteins in clusters 6 and 7 are depicted.

Source data are provided as a Source Data file.

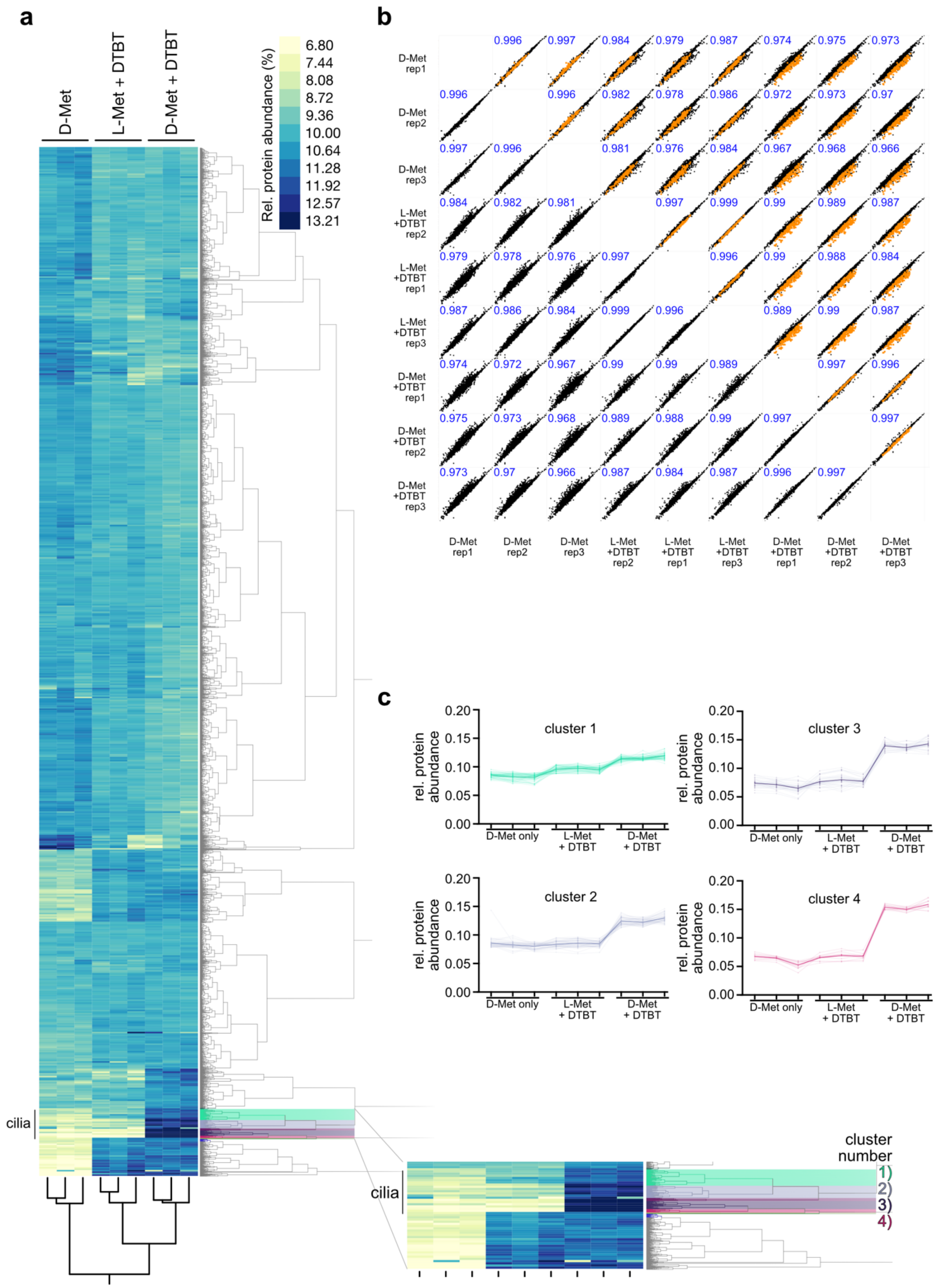

Supplementary Fig. 5

Supplementary Fig. 5: Hierarchical clustering of cilia-iAPEX proteomics in NIH/3T3 cells identifies cilia proteins.

(a) Relative protein abundances (rows) of the individual samples (columns) from NIH/3T3 cilia-iAPEX proteomics experiment (see **Fig. 5a**) were analyzed by two-way hierarchical clustering (Ward's method). Clusters containing known cilia proteins are indicated. All quantified proteins are shown. A magnified view of the cilia-enriched clusters is presented.

(b) Pairwise multiscatter plots of all identified proteins in the proteomics dataset from NIH/3T3 cells, generated in Perseus. log_2-transformed, imputed and Z-Score normalized protein abundance values were used (see **Supplementary Data 2**). Pearson correlation coefficients shown in blue. Top right, ciliary cluster hits (see **(c)** and **Fig. 5d**) are highlighted in orange.

(c) Line plots of clusters containing cilia proteins (1-4) depict the relative abundances of all individual protein across samples (thin lines), with thick lines showing average abundances of all proteins within the clusters.

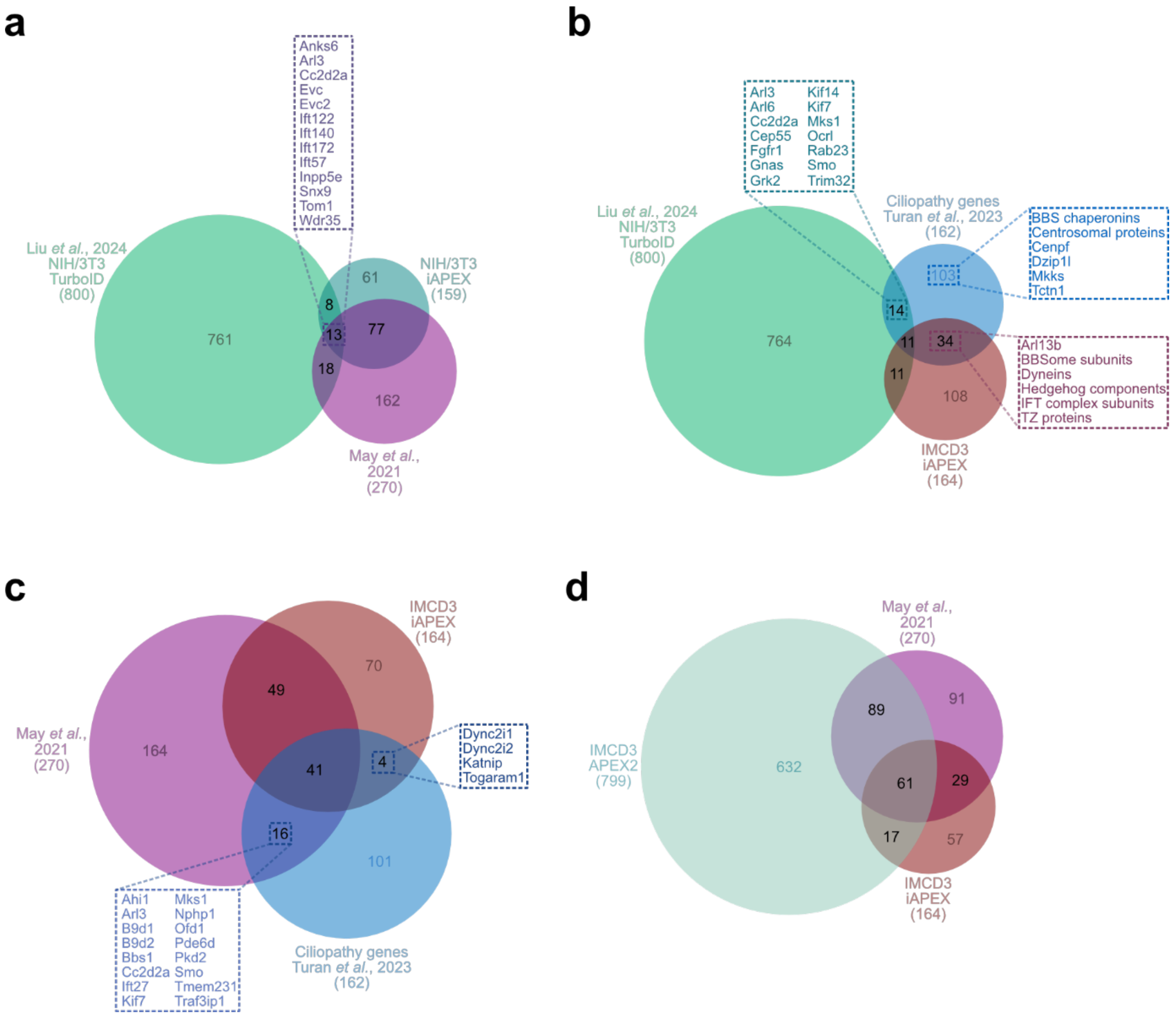

Supplementary Fig. 6: Comparison with other proximity labeling-based proteomic analyses of primary cilia.

(a) Previously reported TurboID-based primary cilia proteome shows limited overlap with cilia-iAPEX proteome. Venn diagram depicts proteomic overlap of iAPEX proximity labeled NIH/3T3 cilia against the TurboID-based cilia proteome from Liu *et al.* and the cilia-APEX2 proteome in IMCD3 cells from May *et al.* Box depicts the proteins present in all three datasets.

(b) cilia-iAPEX proteome has higher coverage of ciliopathy genes than previous studies. Venn diagram of the proteomic overlap between the cilia-iAPEX IMCD3 proteome, the TurboID-based NIH/3T3 cilia proteome and known ciliopathy genes. Boxes show representative proteins.

(c) Venn diagram shows coverage of ciliopathy genes in May *et al.*[17] and this study. Proteins of specific intersections are shown in boxes.

(d) Overlap between APEX-based proteomes in IMCD3 cells. Venn diagram shows intersection between cilia-APEX2 datasets from May *et al.* and this study, compared to the cilia-iAPEX dataset. Source data are provided as a Source Data file.

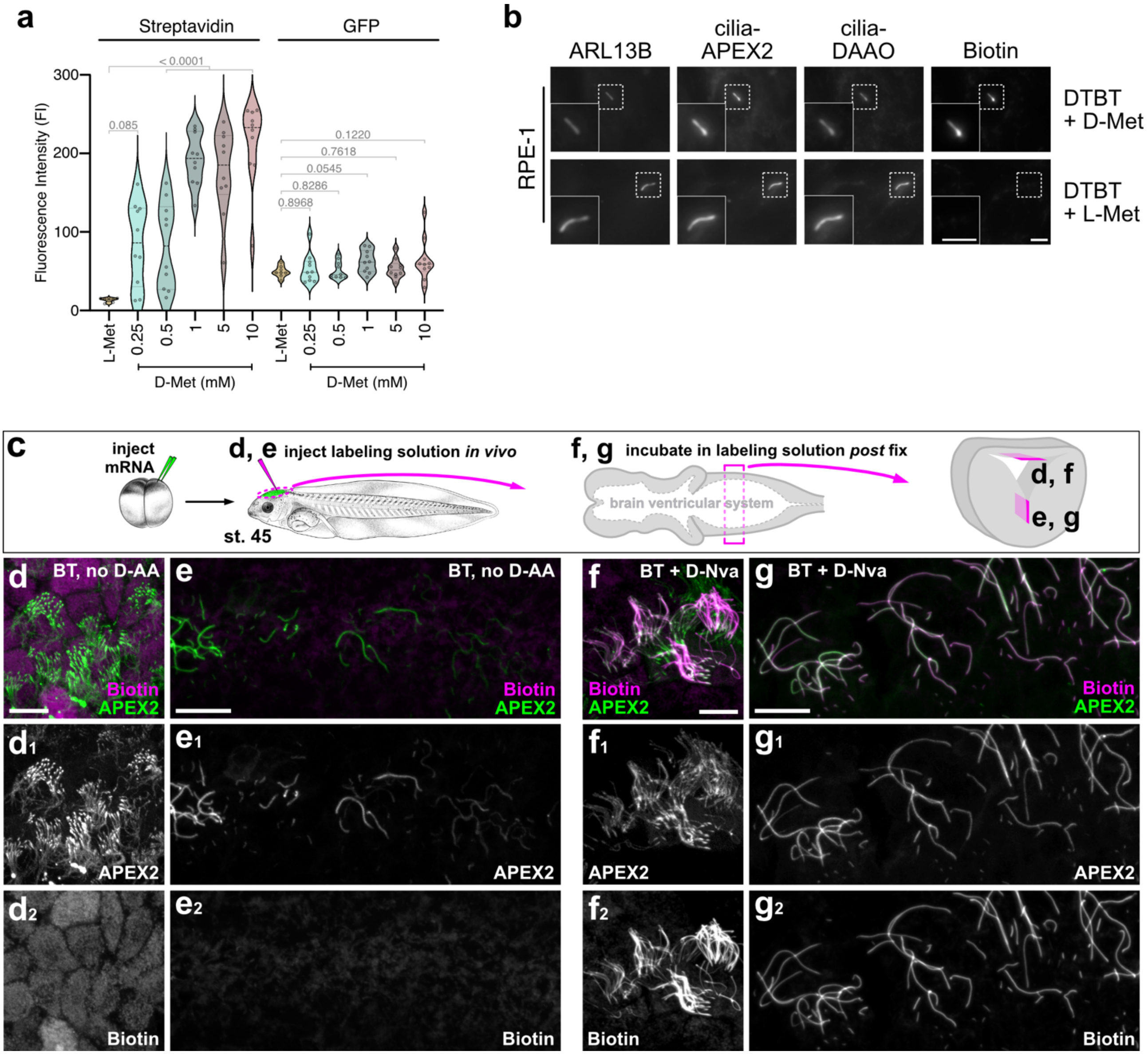

Supplementary Fig. 7

Supplementary Fig. 7: Cilia-iAPEX RPE-1 cell line and *Xenopus laevis* iAPEX labeling after fixation.

(a) Quantification of ciliary tip biotin and GFP-APEX2-GLI2 fluorescence signals from micrographs in **Fig. 6a** are shown. Biotin signal intensities of ciliary tips, defined by GFP signals, were measured. Quartiles and medians are represented by dotted and dashed lines, respectively. Data were analyzed using two-tailed Mann-Whitney test. n = 10; *p* values are indicated. Source data are provided as a Source Data file.

(b) An RPE-1 cell line was infected with lentiviral vectors to express the cilia-iAPEX transgenes (depicted in **Fig. 7a**) and GFP-positive cells sorted by FACS. The resulting cilia-iAPEX RPE cell line was deprived of growth medium for 48 h to induce ciliation, followed by proximity labeling with desthiobiotin tyramide (DTBT) and D-Met or L-Met as indicated (n = 5 independent experiments). Fixed cells were immunostained using antibodies specific to ARL13B and ALFA tag (cilia-DAAO). Cilia-APEX2 was visualized by GFP fluorescence and biotin by fluorescently labeled streptavidin. Scale bars = 5 µm.

(c) mRNA transcribed from plasmid encoding cilia-APEX2 and $PKHD1^{CTS}$-DAAO was injected into one or two dorsal animal blastomeres of four-to-eight-cell *Xenopus laevis* embryos to target constructs to the central nervous system. For post fixation labeling, tadpole stage (st. 45) brains were dissected and incubated in labeling solution. For *in vivo* proximity biotinylation, labeling solutions were injected into the brain ventricular system at st. 45. Brain preparations were (immuno)stained to visualize APEX2 and biotinylation using anti-GFP antibody and fluorescently labeled streptavidin, respectively, in dorsal multiciliated **(d, f)** and ventral monociliated **(e, g)** cells.

(d, e) Control of *in vivo* proximity labeling, ventricle injection of biotin tyramide (BT) without D-amino acid (D-AA) was incubated for 30 min. N = 2 independent experiments, n = 4 embryos analyzed.

(f, g) After 20 min fixation of whole embryos, brains were dissected and incubated in BT and D-Norvaline (D-Nva) for 3 min, followed by quenching / post-fixation. N = 2, n = 11.

Scale bars in **(d – g)**: 10 µm.

Xenopus illustrations © Natalya Zahn (2022).

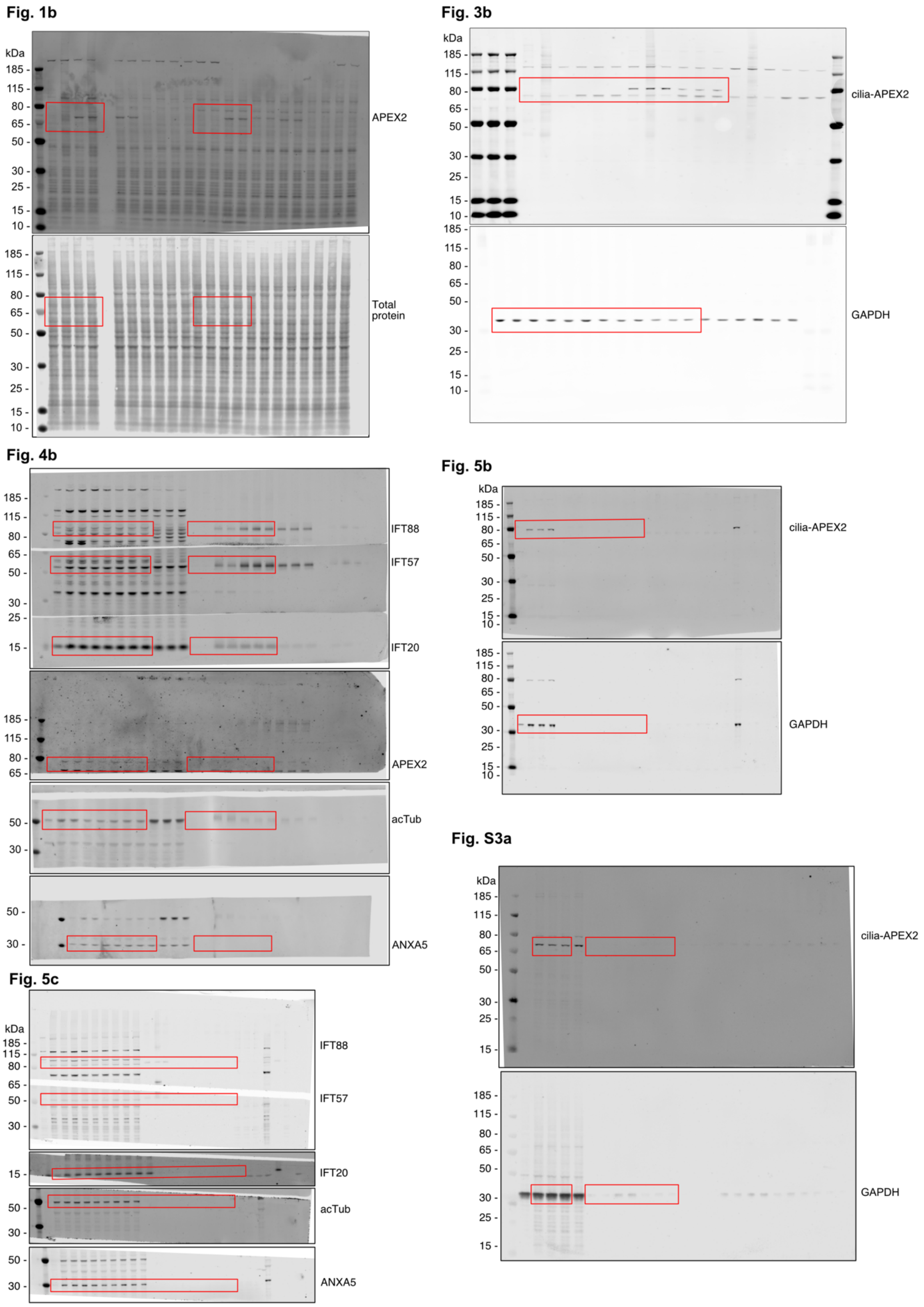

Supplementary Fig. 8: Uncropped immunoblots.

Red boxes indicate areas used for cropped panels in figures.
