# Supplementary material for: An enzymatic cascade enables sensitive and specific proximity labeling proteomics in challenging biological systems
Source: Nat Commun. 2025 Nov 3;16:9691. doi: 10.1038/s41467-025-65405-8 (PMC12583597; doi:10.1038/s41467-025-65405-8)
Supplement: Supplementary file 2 — Description of Additional Supplementary Information [file 41467_2025_65405_MOESM2_ESM.pdf]

Description of Additional Supplementary Files

File Name: Supplementary Movie 1
Description: **Intraflagellar transport in IFT38-GFP-APEX2 and cilia-DAAO expressing IMCD3 cells**
Live-cell imaging of IFT trains in untreated IMCD3 cells co-expressing IFT38-GFP-APEX2 and cilia-localized DAAO. GFP fluorescence was imaged every second for 2 mins. 20 frames per second are shown. Scale bar = 5µm.

File Name: Supplementary Movie 2
Description: **IFT after 30 min of H_2O_2 production in primary cilia**
Live-cell imaging of IFT38-GFP-APEX2 in cells co-expressing cilia-localized DAAO after 30 min of incubation in 10 mM D-Met. Imaging GFP fluorescence for every second for 2 mins. 20 frames per second. Scale bar = 5µm.

File Name: Supplementary Movie 3
Description: **IFT after 30 min of iAPEX labeling in primary cilia**
Live-cell imaging of IFT38-GFP-APEX2 in cells co-expressing cilia-localized DAAO after 30 min of incubation with biotin tyramide and 10 mM D-Met to induce iAPEX labeling in primary cilia. GFP fluorescence was detected every second for 2 mins. 20 frames per second. Scale bar = 5µm.

File Name: Supplementary Movie 4
Description: **H_2O_2 addition results in burst of peroxidase activity throughout the cell**
Live-cell imaging of cilia-iAPEX expressing IMCD3 cells. 50 µM AmUR and 10 mM H_2O_2 were added when indicated. Time lapse shows resorufin fluorescence. Stills show cilia-APEX2 (detected by GFP fluorescence) and resorufin fluorescence before and after substrate addition. (related to **Fig. 3c**).

File Name: Supplementary Movie 5
Description: **D-Met-mediated DAAO activation results in specific peroxidase activity in cilia**
Live-cell imaging of cilia-iAPEX expressing IMCD3 cells. 50 µM AmUR and 10 mM D-Met were added when indicated. Time lapse shows resorufin fluorescence. Stills show cilia-APEX2 (detected by GFP fluorescence) and resorufin fluorescence before and after substrate addition. (related to **Fig. 3d**).

File Name: Supplementary Data 1
Description: **cilia-iAPEX proteomics of IMCD3 primary cilia**
First tab 'Full IMCD3 Dataset' lists the entire dataset from experiment, as depicted in **Fig. 4a**. Samples from mislocalized cyto-iAPEX cell lines were included in 10plex TMT experiment. UniProt identifiers, Gene names and protein descriptions according to the *Mus musculus* proteome database (UP000000589, ID10090, date: 27.10.2022) are listed. Column D shows number of unique quantified peptides for each protein. RAW TMT reporter intensities were summed in Column O to calculate relative TMT protein abundances. Imputed data marked in orange. Columns Z and AA display log_2-transformed average TMT ratios and *p* values of cilia-iAPEX over *Cep164*$^{-/-}$ control, respectively. Columns AB and AC display log_2-transformed

average TMT ratios and *p* values of cilia-APEX2 over control, respectively. *p* values were calculated by two-sided Student's *t* test.
Second tab 'Extracted Cilia Clusters" displays data for proteins in cilia clusters shown in **Fig. 4d**. Colors highlight individual clusters as in **Fig. S4**. Average column means were calculated and displayed in **Fig. S4b**.
Third tab 'Extracted Background Clusters" displays data for proteins in background clusters, parts of which are shown in **Fig. 4f**. Average column means were calculated for **Fig. S4c**.
Fourth tab 'Legend' explains columns in other tabs.

File Name: Supplementary Data 2
Description: **cilia-iAPEX proteomics of NIH/3T3 primary cilia**
First tab 'Full NIH 3T3 Dataset' lists the entire dataset from experiment depicted in Fig. 5a. Samples from cilia-iAPEX NIH/3T3 cells treated with D-Met (no BT control), BT+L-Met (L-Met control) and BT+D-Met (iAPEX) were included in a 10plex TMT experiment. UniProt identifiers, Gene names and protein descriptions according to the *Mus musculus* proteome database (UP000000589, ID10090, date: 27.10.2022) are listed. Column D shows number of unique quantified peptides for each protein. TMT reporter intensities were Z-score column normalized in Columns O to X and summed in Column Y to calculate relative TMT protein abundances. Imputed data was marked in orange. Columns AJ and AK display log_2-transformed average TMT ratios and *p* values of BT+D-Met over D-Met samples, respectively. Columns AL and AM display log_2-transformed average TMT ratios and *p* values of cilia-APEX2 over control, respectively. *p* values were calculated by two-sided Student's *t* test.
Second tab 'Extracted Cilia Clusters" displays data for proteins in cilia clusters shown in Fig. 5d. Colors highlight individual clusters as in **Fig. S5**. Average column means were calculated and displayed in **Fig. S5c**.
Third tab 'Legend' explains columns in other tabs.

File Name: Supplementary Data 3
Description: **Comparison of cilia-iAPEX proteomics to cilia-APEX2**
First tab 'May *et al.*, 2021' lists gene names of the cilia-APEX2 proteome as defined in May *et al.*, 2021.
Second tab 'IMCD3 cilia-iAPEX' lists proteins identified in three cilia clusters from IMCD3 cilia-iAPEX proteomics (**Fig. 4d** and **Table S1**).
Third tab 'NIH-3T3 cilia-iAPEX' lists gene names of proteins identified in four cilia clusters from cilia-iAPEX proteomics in NIH/3T3 cells (selected clusters shown **Fig. 5d** and **Table S2**).
Fourth tab 'Venn diagram' lists gene names in the individual Venn diagram sets and intersections (**Fig. 5e**) as indicated.

File Name: Supplementary Data 4
Description: **Lists of reagents**
Antibodies, plasmids, oligonucleotides and cell lines used in this study are listed in separate tabs.
