# Supplementary material for: An enzymatic cascade enables sensitive and specific proximity labeling proteomics in challenging biological systems
Source: Nat Commun. 2025 Nov 3;16:9691. doi: 10.1038/s41467-025-65405-8 (PMC12583597; doi:10.1038/s41467-025-65405-8)
Supplement: Supplementary file 12 — Reporting Summary [file 41467_2025_65405_MOESM12_ESM.pdf]

nature portfolio

Corresponding author(s): David U. Mick

Last updated by author(s): Oct 9, 2025

Reporting Summary

Nature Portfolio wishes to improve the reproducibility of the work that we publish. This form provides structure for consistency and transparency in reporting. For further information on Nature Portfolio policies, see our Editorial Policies and the Editorial Policy Checklist.

Statistics

For all statistical analyses, confirm that the following items are present in the figure legend, table legend, main text, or Methods section.

n/a	Confirmed	
☐	☒	The exact sample size (*n*) for each experimental group/condition, given as a discrete number and unit of measurement
☐	☒	A statement on whether measurements were taken from distinct samples or whether the same sample was measured repeatedly
☐	☒	The statistical test(s) used AND whether they are one- or two-sided<br>*Only common tests should be described solely by name; describe more complex techniques in the Methods section.*
☒	☐	A description of all covariates tested
☐	☒	A description of any assumptions or corrections, such as tests of normality and adjustment for multiple comparisons
☐	☒	A full description of the statistical parameters including central tendency (e.g. means) or other basic estimates (e.g. regression coefficient) AND variation (e.g. standard deviation) or associated estimates of uncertainty (e.g. confidence intervals)
☐	☒	For null hypothesis testing, the test statistic (e.g. *F*, *t*, *r*) with confidence intervals, effect sizes, degrees of freedom and *P* value noted<br>*Give P values as exact values whenever suitable.*
☒	☐	For Bayesian analysis, information on the choice of priors and Markov chain Monte Carlo settings
☐	☒	For hierarchical and complex designs, identification of the appropriate level for tests and full reporting of outcomes
☐	☒	Estimates of effect sizes (e.g. Cohen's *d*, Pearson's *r*), indicating how they were calculated

Our web collection on statistics for biologists contains articles on many of the points above.

Software and code

Policy information about availability of computer code

Data collection	All mass spectrometry data were collected using FragPipe.
Data analysis	Graphpad Prism (10.3.1(464))<br>JMP software (Statistical Analysis System; v17.2.0)<br>Perseus (version 2.0.11.0)

For manuscripts utilizing custom algorithms or software that are central to the research but not yet described in published literature, software must be made available to editors and reviewers. We strongly encourage code deposition in a community repository (e.g. GitHub). See the Nature Portfolio guidelines for submitting code & software for further information.

Data

Policy information about availability of data

All manuscripts must include a data availability statement. This statement should provide the following information, where applicable:

- Accession codes, unique identifiers, or web links for publicly available datasets
- A description of any restrictions on data availability
- For clinical datasets or third party data, please ensure that the statement adheres to our policy

Data of this study are available in the Figures or the Supplementary Information. All proteomics data generated in this study have been deposited to the ProteomeXchange Consortium via the PRIDE partner repository under accession code PXD060138[http://proteomecentral.proteomexchange.org/cgi/GetDataset?ID=PXD060138]. Further source data are provided with this paper.

Research involving human participants, their data, or biological material

Policy information about studies with human participants or human data. See also policy information about sex, gender (identity/presentation), and sexual orientation and race, ethnicity and racism.

Reporting on sex and gender	No human participants were involved in this study
Reporting on race, ethnicity, or other socially relevant groupings	No human participants were involved in this study
Population characteristics	No human participants were involved in this study
Recruitment	No human participants were involved in this study
Ethics oversight	No human participants were involved in this study

Note that full information on the approval of the study protocol must also be provided in the manuscript.

Field-specific reporting

Please select the one below that is the best fit for your research. If you are not sure, read the appropriate sections before making your selection.

☒ Life sciences ☐ Behavioural & social sciences ☐ Ecological, evolutionary & environmental sciences

For a reference copy of the document with all sections, see nature.com/documents/nr-reporting-summary-flat.pdf

Life sciences study design

All studies must disclose on these points even when the disclosure is negative.

Sample size	Tandem-mass-tag (TMT)-based quantitative proteomics is based on isobaric tags, which allow multiplexing of a finite amount of samples, in our case 10. Our experiments required the analysis of three different conditions in one (max 10plex) experiment, such that relevant samples were analyzed in triplicates. In one instance, technical control samples with small variation were analyzed in duplicates.
Data exclusions	TMT MS experiments were 10plex. MS data of all samples have been analyzed (without exclusions) and can be found in supplementary tables. However, only relevant samples have been further analyzed by hierarchical clustering and other methods, as displayed in the Figures. Therefore, for IMCD3 cilia proteomics experiments, the data from three irrelevant samples (different condition not to be analyzed) have been excluded in the final analysis. Similarly, for the 10plex TMT cilia proteomics experiment in NIH/3T3 cells, the two non-labeling conditions were performed in triplicate, while labeled samples in quadruplicate. For consistency, one replicate was randomly chosen and excluded to compare triplicates for all conditions. The original data for the excluded replicate is shown in Supplementary Data 2.
Replication	All experiments were replicated at least twice (for technical confirmations) or more often when technical or biological variations were analyzed, as indicated throughout the manuscript. For Figs. 5g, 6b, 7b, 7c results were replicated with slight experimental adjustments and number of replicates are, therefore, indicated as n = 1.
Randomization	For TMT MS experiments, in which quadruplicate samples were analyzed, three samples were randomly chosen, as described in data exclusion. For experiments where individual cilia have been analyzed by microscopy, images were taken and chosen randomly for subsequent analysis. Specific cilia to be analyzed in an unbiased fashion were from randomly chosen, representative images by identification through segmentation software (CiliaQ, see methods section).
Blinding	For MS analysis, group allocation was not provided prior to analysis.

Reporting for specific materials, systems and methods

We require information from authors about some types of materials, experimental systems and methods used in many studies. Here, indicate whether each material, system or method listed is relevant to your study. If you are not sure if a list item applies to your research, read the appropriate section before selecting a response.

Materials & experimental systems

n/a	Involved in the study
☐	☒ Antibodies
☐	☒ Eukaryotic cell lines
☒	☐ Palaeontology and archaeology
☐	☒ Animals and other organisms
☒	☐ Clinical data
☒	☐ Dual use research of concern
☒	☐ Plants

Methods

n/a	Involved in the study
☒	☐ ChIP-seq
☒	☐ Flow cytometry
☒	☐ MRI-based neuroimaging

Antibodies

Antibodies used	acTub (6-11B-1) Sigma-Aldrich (Merck) T7451<br>ALFA sdAb rabbit-fc NanoTag Biotechnologies N1583<br>ALFA sdAb mouse-fc NanoTag Biotechnologies N1582<br>Annexin V Proteintech 66245-1-Ig<br>ARL13B Proteintech 17711-1-AP<br>FLAG (M2) Sigma-Aldrich (Merck) F1804<br>GAPDH (1E6D9) Proteintech 60004-1-Ig<br>GFP (3H9) Proteintech (Chromotek) 3h9<br>IFT20 Proteintech 13615-1-AP<br>IFT57 Proteintech 11083-1-AP<br>IFT88 Proteintech 13967-1-AP<br>S-tag Thermo PAS-81631
Validation	Antibodies were either vailidated in the study or have been validated extensively in existing literature, as can be accessed by the providers.

Eukaryotic cell lines

Policy information about cell lines and Sex and Gender in Research

Cell line source(s)	IMCD3 FlpIn (Wright et al., 2011; 10.1101/gad.173443.111); NIH/3T3 (gift from P. Niewiadomski; Niewiadomski et al., 2015; 10.1007/978-1-4939-2772-2_11); 3T3-L1 (ATCC, CRL-1772); C2C12 (ATCC); RPE1 FRT/TO (gift from J. Mansfeld; Zerjatke et al., 2017; 10.1016/j.celrep.2017.05.022); HeLa Kyoto (Schrul and Kopito, 2016; 10.1038/ncb3373).<br>No primary cell lines were derived.
Authentication	Cell lines were authenticated by immunohistochemistry using specific markers, and by proteomic analysis (IMCD3, NIH-3T3, RPE1), as well as karyotyping (for IMCD3 and NIH-3T3 cells).
Mycoplasma contamination	All cell lines were regularly tested to be negative for mycoplasma.
Commonly misidentified lines<br>(See ICLAC register)	N/A

Animals and other research organisms

Policy information about studies involving animals; ARRIVE guidelines recommended for reporting animal research, and Sex and Gender in Research

Laboratory animals	Xenopus laevis
Wild animals	The study did not involve wild animals.
Reporting on sex	Sex was not considered in study design.
Field-collected samples	The study did not involve samples collected from the field.
Ethics oversight	Regional government Stuttgart, Germany

Note that full information on the approval of the study protocol must also be provided in the manuscript.

Plants

Seed stocks	N/A
Novel plant genotypes	N/A
Authentication	N/A
